# Graduate perceptions of their interprofessional practice: Lessons for undergraduate training

**DOI:** 10.4102/phcfm.v16i1.4706

**Published:** 2024-12-18

**Authors:** Jana Müller, Elize Archer, Ian Couper

**Affiliations:** 1 Division of Rural Health (Ukwanda), Department of Global Health, Faculty of Medicine and Health Sciences, Stellenbosch University, Cape Town, South Africa; 2 Department of Health Professions Education, Faculty of Medicine and Health Sciences, Stellenbosch University, Cape Town, South Africa

**Keywords:** interprofessional education, interprofessional collaborative practice, undergraduate, rural, clinical training

## Abstract

**Background:**

Interprofessional education (IPE) during undergraduate training (UGT) is considered important for new graduates to collaborate inter-professionally. There are, however, well-documented workplace challenges that hinder their involvement in interprofessional collaborative practice (IPCP) such as professional hierarchy, poor role clarification and communication challenges.

**Aim:**

This article explores graduates’ perceptions of the value rural undergraduate IPE had on their IPCP during their first year of work.

**Setting:**

Graduates were based in seven different provinces of South Africa ranging from tertiary-level institutions to community day clinics.

**Methods:**

A qualitative case study was conducted with 16 first-year graduate participants from 5 different health professions who participated in undergraduate IPE while placed on a rural platform. Individual semi-structured interviews were conducted in 2023.

**Results:**

Key factors related to UGT that facilitated IPCP during graduates’ first year of work were interprofessional relationship development, practice-based IPE and the focus on holistic patient-centred care. Graduates were, however, challenged by self-doubt, communication barriers and hierarchy in the workplace. Their recommendations for undergraduate IPE included role modelled and contextually relevant interprofessional skills development, practical advocacy and communication training, longer placements or shared learning spaces.

**Conclusion:**

Findings indicate that IPE during undergraduate rural clinical placements promotes interprofessional relationship development that extends into the workplace after graduation. However, IPE must be role modelled in the clinical environment and aligned to the reality of the healthcare system for students to develop the skills required to navigate IPCP as graduates.

**Contribution:**

This article offers recommendations for responsive undergraduate IPE to promote IPCP after graduation.

## Introduction

Interprofessional collaborative practice (IPCP), where different healthcare professionals work together with the patient to produce better healthcare services, has been shown to improve patient care, job satisfaction and healthcare systems.^1,2,3^ However, there are multiple challenges that healthcare workers face that hinder the success of IPCP both internationally^3^ and in Africa.^4^ The barriers and facilitators to effective collaboration are different across various healthcare systems and can be found at the organisational, team and individual level.^1,3,4^ Wei et al.^3^ found that workplace values, culture, support structures, hierarchy, leadership, role clarity, communication, trust and respect, among others, play a role in effective IPCP. In Africa, additional barriers to IPCP such as discrepancies in salary, heavy workload and insufficient human resources were identified.^1,4^

One of the recommended strategies to overcome some of the obstacles to IPCP in the workplace is to introduce interprofessional education (IPE) into undergraduate training (UGT).^5,6^ Interprofessional education takes place when students from two or more professions learn about, with and from one another to improve collaboration and quality of care and services.^5^ Despite the introduction of IPE into student training, however, graduates still report challenges with implementing IPCP in the workplace.^7,8^ In fact, graduates who work in environments where interprofessional teamwork is not commonplace or prioritised are unlikely to engage in IPCP despite undergraduate IPE.^9^

In South Africa (SA), studies have found that there is a limited understanding of what IPCP is among healthcare workers who struggle to differentiate between multidisciplinary collaboration (professionals from the same discipline, e.g., rehabilitation, working together) versus interprofessional collaboration.^4^ There is also a clear indication that healthcare workers are not familiar with the roles of their teammates and still battle with interprofessional communication challenges.^4,10^ Many professionals also question the value of IPCP.^11^ A culture of hierarchy has been identified as the overriding team barrier that affects whether healthcare workers believe IPCP could be successful in the places where they work.^4,11^ In addition to these challenges, patient turnover, poor infrastructure, inadequate space and staff shortages have also been reported as barriers to IPCP in SA.^4^

Although IPE has been introduced in approximately 50% of African universities offering health professions education (HPE),^12^ training programmes must be cognisant of the multitude of predicted barriers to IPCP that their graduates will face. Paradis and Whitehead^13^ caution that IPE without exposure to power dynamics and structural and organisation barriers will limit the value it has in promoting IPCP. There is a need for HPE to be purposively aligned with the needs of the healthcare system;^14^ and training should mimic the reality thereof. A lack of alignment will negatively affect patients and the healthcare professionals working there.^14^ A philosophical, contextual and practical alignment between IPE and the needs and challenges of the healthcare system is needed to produce graduates who are capable of engaging in IPCP.^14,15,16^

To develop responsive IPE curricula that are inline with the needs of the healthcare system, educators need to understand what interprofessional learning experiences graduates perceive as valuable for collaborative practice. Exploring and documenting the first-hand experiences and recommendations of graduates who have undergraduate interprofessional exposure will enhance interprofessional learning and teaching in undergraduate curricula.

This article sets out to explore the influence of undergraduate interprofessional learning experiences on the ability of graduates to collaborate inter-professionally. For the purposes of this study, graduates were followed-up at the end of their first year of work to understand IPCP in the South African public healthcare context.

### Background

At Stellenbosch University, SA, the Faculty of Medicine and Health Sciences has no structured IPE curriculum for the various undergraduate programmes. There have, however, been stand-alone IPE activities taking place in various contexts.^17,18,19^ For the last 12 years, a practical approach to IPE called the Collaborative Care Project (CCP) was introduced to final-year students from five healthcare professions who were placed in a rural town in SA. This project, which takes place weekly, has been previously described.^20^ The CCP includes students from human nutrition, medicine, occupational therapy, physiotherapy and speech, language and hearing therapy. Because of positive feedback, the CCP concept was expanded in 2019 to another rural training site, where students from more than one profession train, in a different province of SA.^21^ As part of the project, students present cases related to patients they are busy seeing on the platform and an interprofessional discussion is facilitated by a lecturer using the International Classification for Function, Disability and Health (ICF). The ICF is considered an effective tool in promoting IPE and IPCP.^22,23^ Students are required to engage in holistic evaluation of, and management planning for, the patient presented. Where possible, student teams visit patients and their families in their homes, at the local clinic or at the bedside in the hospital, to include them in the interprofessional team as per the definition of IPCP.^24^ Apart from the CCP, students from various professions stay together in shared residences and work in the same healthcare facilities on the two rural platforms; thus they also have opportunities to engage with each other inside and outside of the working environment.

A previous paper exploring students’ perceptions of their interprofessional learning experiences on these two rural training platforms and their engagement in IPCP formed part of Phase 1 of J.M.’s PhD.^25^ Phase 2 of this study explored the perceptions of these same participants regarding their engagement in IPCP in the workplace as graduates. This article explores these graduates’ perceptions of IPCP during their first year of work and what recommendations they have for undergraduate HPE.

## Methodology

### Study design

A qualitative explanatory multiple case study research design^26^ was adopted to explore the perceived value of interprofessional experiences on graduates’ IPCP. Explanatory research can assist with identifying emerging causal links^27^ between undergraduate interprofessional learning experiences and graduates’ perceived ability to collaborate inter-professionally during their first year of work. Multiple case study designs are recommended as a means of data triangulation to improve the transferability, not of the site or population, but of the theory.^26,28^ The investigation and analysis of multiple cases in this research, where every research participant is considered a case, aimed to understand the experience of undergraduate IPE on first-year graduates’ IPCP in SA.

### Study population and sampling

Participants were recruited during 2022 as part of Phase 1 of J.M.’s PhD study from all final-year undergraduate students who had experienced longitudinal multidisciplinary placement (8–40 weeks) in either of the two rural towns in 2022. Students were contacted via email and WhatsApp by their academic coordinators with an invitation to participate in this study. Sixteen purposively selected participants from Phase 1 included two students each from the following degree programmes: Medicine, Physiotherapy, Occupational Therapy, Human Nutrition and Speech Language and Hearing therapy from both rural training sites. A more detailed description of the methodology has been published previously.^25^

All 16 participants were completing their first year of work during 2023 in the South African public healthcare sector across seven of the nine provinces.

### Data collection

The 16 purposively selected participants from 2022 were contacted telephonically in 2023 and invited to participate in individual follow-up interviews, and suitable dates, times and places were arranged. These graduates were interviewed face-to-face by J.M. using a semi-structured interview guide (Box 1). Interviews, which lasted between 60 min and 90 min were recorded, transcribed and anonymised by J.M. using pseudonyms known only to her.

BOX 1 Semi-structured interview guide for graduate participants.

**Semi-structured interview guide**

1. Where have you been working this year? How has it been?
2. What is your understanding of collaborative patient care?
3. What is your experience of collaborative patient care where you have been working?
4. Describe some of the experiences you had of working or engaging with professionals from other disciplines this year?
5. How would you perceive your ability to collaborate professionally to improve patient care and health systems during this year?
6. You mentioned …………………… last year during our interview as a …………… to being able to work collaboratively with students from other professions. What do you think about those experiences now in hindsight?
7. What aspects of your undergraduate training stand out as having been of greatest benefit in terms of your preparedness to manage patients collaboratively?
8. What aspects of working inter-professionally to improve patient health did you feel least prepared for during this year?
9. Looking back, what do you think could have been different during your undergraduate training to better equip you to work inter-professionally to improve collaborate patient care as a healthcare professional in South Africa?

### Analysis

An in-depth reflexive thematic analysis^29^ was done by J.M. who conducted a broad sweep analysis of interview data by listening, transcribing and reading each interview transcript after which a preliminary code list was developed. An independent qualitative researcher Innocentia Lediga (I.L.) also developed her own code lists and a final code list for data analysis was agreed upon and discussed with I.C. and E.A. J.M. coded every transcript using ATLAS.Ti software^30^ and generated themes based on the shared meaning of the codes.^29^ These data were reviewed with E.A., I.C. and I.L., and the final findings were then developed.

### Positionality and trustworthiness

E.A., I.C. and J.M. are from different healthcare professions, but from similar demographic backgrounds; therefore, I.L. who is from a different ethnic, cultural and professional background was also involved in an independent analysis of the data and to engage in active reflection with J.M. This was done to deepen the interpretation of the data and understanding of the diversity of experiences of graduates with differing demographic profiles.

J.M. has been involved in the CCP for 12 years and in the establishment and research of IPE on the two rural training platforms included in this study. I.C. as the head of the Division of Rural Health (Ukwanda) has an overarching view of the academic offerings on the two rural training platforms that form part of this study and has been involved in much of the previous research. E.A., from the Department of Health Professions Education, has keen insight into the theoretical and practical implementations of HPE. I.L. had no prior involvement with CCP or the students at the rural training platforms during 2022.

J.M.’s existing interaction and relationship with the participants as students in 2022 facilitated a deeper exploration of experiences and iterative processes to data collection. J.M. could recognise conflicting information and probe or rephrase questions based on the previous interviews held in 2022 providing a more comprehensive understanding of the research data.^31,32^ The involvement of an additional researcher (I.L.) to assist with the process of data analysis provided insight into the data from a different perspective and provided an opportunity for reflection^32^ and a chance to enhance the credibility and confirmability of our research findings.^33^

### Ethical considerations

Ethical approval to conduct the study was granted by the Faculty of Medicine and Health Sciences Health Research Ethics Committee (Reference S22/04/069), the Stellenbosch Division of Institutional Research and Planning and the Committee for Undergraduate Teaching. Interviewees participated voluntarily after providing informed consent.

## Results

All 16 participants were first-year graduates of Stellenbosch University working in both rural (*n* = 10) and urban (*n* = 6) areas of SA. Eleven participants were female and five were male (based on their university records); nine participants reported being Afrikaans first language speakers, and the remaining seven represented six different first languages. The participants were stationed at tertiary, secondary or district level hospitals or at district clinics across seven of the nine provinces of SA (Table 1).

**TABLE 1:** Details of the number of participant’s and their placement sites during their first year of work.

Study by profession	Number of graduates	Health service level	Number of graduates	Provinces	Number of graduates
HN¶	2	District clinic (DC)	1	Eastern Cape	1
MBChB‡	4	District Hospital (DH)	8	Free State	3
OT†	4	Provincial Hospital (PH)	5	Gauteng	3
PT*	4	Tertiary Hospital (TH)	2	Kwa-Zulu Natal	2
SLT§	2	-	-	Northern Cape	5
-	-	-	-	Northwest	1
-	-	-	-	Western Cape	1

* , Physiotherapy;

† , Occupational therapy;

‡ , Medicine;

§ , Speech, language and hearing therapy (two participants were based at both rural training platforms as undergraduate students);

¶ , Human Nutrition (participants were only based at one rural training platform as undergraduate students hence there being only two of them).

In the following section, we present the data under three themes: (1) the perceived value of undergraduate IPE; (2) challenges to IPCP in the workplace; and (3) recommendations for undergraduate HPE. Themes and sub-themes generated in this article are presented in Table 2. The participants’ quotes are linked to pseudonyms, their profession and healthcare service level where they worked (for abbreviations used, see Table 1).

**TABLE 2:** Themes and sub-themes.

Themes	Sub-themes
1. Perceived value of undergraduate interprofessional education	1.1 Practical exposure 1.2 Developing a patient-centred interprofessional collaborative practice philosophy
2. Challenges to interprofessional collaborative practice in the workplace	2.1 Seniority and hierarchy 2.2 Professional hierarchy and role clarification 2.3 Understanding context and culture
3. Recommendations for undergraduate health professions education	3.1 More contextually relevant exposure 3.2 Communication, advocacy and role modelling in practice 3.3 Accepting differences 3.4 The value of relationships

### Theme 1: Perceived value of undergraduate interprofessional education

#### Sub-theme 1.1: Practical exposure

Participants noted that their previous practical IPE exposure on the rural platform influenced their expectations of IPCP in the workplace. Bulumko commented that having the responsibility of working in a team and knowing the role of his teammates during UGT was not something he witnessed during his first year of work:

‘The nice thing about being in [*X rural training site*], you feel a sense of responsibility to know what does your team do and you feel like you’re part of a team. Unlike what’s happening here [*in current health care facility*] … They don’t even know what the physio is going to do.’ (Bulumko, MBChB, PH)

Despite IPCP not being commonplace where many graduates worked, their undergraduate experiences helped them see how patient care could and should be:

‘The emphasis in our curriculum that they put on, collaboration within the clinical setting that you’re placed is also something that definitely prepared me for it [*interprofessional collaboration*].’ (Nicolette, OT, DH)

This encouraged graduates to connect with other professionals based on their insight into what these professionals had to offer patients:

‘Last year we did get exposure to it [*IPE*]. So I’m very thankful because if I see a stroke patient my first thought is, Oh, refer to the speech so that she can help to assess the swallowing. And I think if we didn’t have that exposure last year, I maybe would have tried to investigate by myself … I think you can get so into your own mindset that you forget, but what about all of these other people?’ (Carieke, HN, DH)

Having a better understanding of the value of a team approach prompted Andisa, a doctor, to get involved in an existing multidisciplinary rehabilitation team at the hospital where she worked:

‘Because of my foundation in [*X rural training site*] I’d really like to try to finish my discharge early, do all my blood so that when the time comes, I’m able to go to the MDT meeting. It was beautiful and I’m really so grateful for it. For [*X*] for laying that foundation for me.’ (Andisa, MBChB, TH)

Nicolette described how her interaction with other professionals in the workplace appeared to have effected a change within the team dynamic leading to more patient referrals and communication:

‘So, we started to attend the doctor’s meetings on a Monday morning … so that they are aware of our [*occupational therapy*] presence. And when they discuss patients, we advocate for them to refer to us. So that’s also been something that’s improved the quality of care … We’ve been receiving more referrals.’ (Nicolette, OT, DH)

Nicolette further reflected that her desire for patient-centred IPCP prompted her to advocate for comprehensive and collaborative care:

‘In the beginning, because I think the doctors often they have a time when they need to discharge patients, [*I was*] telling them like, “for the sake of rehab, … what is your plan? What are you thinking about?”’ (Nicolette, OT, DH)

#### Sub-theme 1.2: Developing a patient-centred interprofessional collaborative practice philosophy

The participants insight and drive to collaborate inter-professionally appeared to have been influenced by the comprehensive approach to patient care during UGT, which Mari felt shifted her perspective about engaging with other professions, despite her reservations about doing so:

‘You can have conversations with the different professions, you don’t actually have to be scared to talk to the doctors type thing. Because at the end of the day, it is about the patients.’ (Mari, PT, DH)

The perspective of patient-centred care resulted in Emile realising the importance of advocating for his and other professions in an environment that did not prioritise IPCP:

‘If you think how many patients are going to miss out on having these services [*speech, language and hearing therapy*] if you don’t advocate for the patients. And that changed my whole mindset … to also be adamant and to say what OT does, what physio does, what speech does, what dietetics do is important. And what nursing does is important.’ (Emile, SPT, TH)

This advocacy and intentional engagement of participants with their colleagues led to what graduates perceived to be a shift in IPCP behaviour in their environment and the possibility of improved patient care:

‘So I think initially at the beginning of the year, I’ve had to initiate a lot more. But now, recently, a lot of the doctors, once they want to start thinking about discharging, they do ask [*and*] discuss with physio, “Is it now time to discharge?”’ (Mieke, PT, DH)

Some participants felt that their IPE exposure on the rural platform and IPCP mindset set them apart from graduates from the same university who might not have had the same IPE experience. Anja, a medical graduate who had spent her final year on the rural platform, comments:

‘I think it [*collaborative care*] really helped, and there’s … people [*who did not train on the rural platform*] and there’s … people [*who trained on the rural platform*] So, it’s specific … I had a much better understanding of what I can take to different people.’ (Anja, MBCHB, DH)

Sindiso was stationed at a hospital with graduates from previous years who had also trained on the same rural platform he had and taken part in the CCP learning experience. Despite not knowing these graduates before working at the same hospital, he knew they had similar IPE experiences. He commented:

‘I know they understand the whole culture and what we did … they went to [*X*] in their final clinical years, so they already understand collaborative care. It’s not a conversation that I have to have.’ (Sindiso, OT, DH)

He further explained:

‘I can hear even by the questions during ward round … where the consultants present cases … So you never really get to say anything … They [*the medical graduates from X*] would ask about occupational therapy, “Why haven’t you done a play program?” So I’m like, okay, now you’re including me … and it’s only been them.’ (Sindiso, OT, DH)

Carieke, a dietician, who was not placed at a facility with peers from the rural platform relied on the interprofessional relationships she previously developed during her rural placement to manage her patients holistically:

‘I messaged the OT who was in the pod next to me last year [*in residence*] And I was like “XX do you see SAM [*severe acute malnutrition*] patients for playtime?” She was just like “Oh, yes it’s very crucial for SAM” and she gave me this whole breakdown and I was like, “Oh my word I didn’t know. I’m going to start referring my SAM patients.”’ (Carieke, HN, DH)

### Theme 2: Challenges to interprofessional collaborative practice in the workplace

Although some graduates started new initiatives related to IPCP during their first year, many of them faced challenges such as a lack of interest or a lack of support for these new initiatives. Sindiso reflected on how his colleagues (medical and nursing) were:

‘[*S*]urprised when I even explain[*ed*] the whole domains of OTs, the whole person when I advocated for patient discharge via occupational therapy’. (Sindiso, OT, DH)

He wanted his colleagues to:

‘Just screen [*patients*] before they go home, because I had to explain that environmental modifications are important’. (Sindiso, OT, DH)

Despite this, Sindiso faced the challenge of lack of support:

‘Like today, I get a referral. Tomorrow, I go and check. They’re gone.’ (Sindiso, OT, DH)

#### Sub-theme 2.1: Seniority and hierarchy

The majority of participants perceived the lack of support of their IPCP initiatives to be related to issues of hierarchy. Hierarchy was seen to be related to both professions and the seniority of position both within and outside of graduates’ professional departments:

‘I expected to be working more as a team together. But there’s a very, very high hierarchy above me. A lot of criticism and not really building up, more breaking down.’ (Jeandre, HN, DH)

This experience affected Jeandre inclination to initiate IPCP, especially when the success thereof would be affected by hierarchy and the lack of support from within her own department:

‘I feel adequately prepared to be able to [*collaborate*]. If I necessarily will, it’s another story. Because … I don’t know how, not necessarily successful, but consistent it will be. Like, to initiate something and continuously do it is very difficult without the proper management on your team.’ (Jeandre, HN, DH)

Participants saw their position as new graduates as a barrier to their opinions being accepted or valued by the rest of the team:

‘We’ve got people here … they’re as old as the building, you know, and they’re just like matriarchs in the hospital and they like, “Oh, they’re little commserves [*new graduates*],” you know.’ (Rebecca, PT, DH)

Kari, however, reflected that senior support alone would not be enough to promote IPCP if a culture of professional hierarchy between healthcare workers persisted in the workplace:

‘Senior management advocating for collaborative care of a patient definitely makes a difference in the wards … but if doctors as a whole shut you down, often you won’t be as keen to go and advocate.’ (Kari, OT, PH)

#### Sub-theme 2.2: Professional hierarchy and role clarification

Interprofessional conflict related to hierarchy was fuelled by a perceived lack of understanding of roles and scope of practice by senior colleagues in the workplace. Taugheeda had this experience in high care:

‘The nurses believe that it’s our role to do suctioning. The HPCSA [*Health Professions Council of South Africa*] guidelines for speech therapists does not include suctioning. We try to communicate that but they outright undermine our degree. One of the M.O.s [*medical officers*] have actually said … “then why did you study? If you can’t do this, why did you study?”’ (Taugheeda, SPT, DH)

Based on these perceptions of hierarchy, participants reflected on their hesitation to approach either seniors or people from other professions to initiate IPCP or advocate for their profession:

‘You maybe want to collaborate with them. But they might not feel the same because you are fresh out of university.’ (Nicolette, OT, DH)

‘The undermining, that’s what I’m scared of … I love what I do so much. I want to share what I do. But the attitudes, that’s where I just feel, so discouraged … So it gets difficult.’ (Sindiso, OT, DH)

This hesitancy and insecurity to interact with other professions was especially marked when engaging with doctors. Some participants commented that they felt they were an inconvenience and would not be taken seriously:

‘Occupational Therapy teaches you about affect. So, you can see [*and*] I don’t know if I’m really being taken seriously or not. And that discourages me so much.’ (Sindiso, OT, DH)

Participants reported that doctors at times appeared angry and distracted and in some cases graduates did not communicate despite knowing how important their own profession was to the management of the patient:

‘They [*the doctors*] look angry. I think I’m very sensitive to inconvenience someone. So, if I see that you’re not in the right mind space to take on new information and discuss the patient, then I’m not going to.’ (Mari, PT, DH)

Facing persistent barriers to IPCP, especially when graduates initiated collaboration, resulted in feelings of despondency and getting to the point where they simply wanted the value of their profession to be acknowledged:

‘I think it’s just the thing of if you get enough resistance throughout the years that you practice, it dims a bit your ability to create your own energy and motivation to do that [*collaboration*]. I mean, I actually see it.’ (Mari, PT, DH)

In frustration, Sindiso commented that:

‘[*I*]t’s basically just being seen, being seen for what I do. Even though I want to be seen for who I am, but I want to be seen also for what I do.’ (Sindiso, OT, DH)

#### Sub-theme 2.3: Understanding context and culture

Anja proposed that the presence of hierarchy and a perceived lack of interest could be a result of not having the bigger picture, and not understanding the workplace culture:

‘I think it’s an unconscious response for doctors. I know it is a hierarchy thing … I also don’t always feel I can consult them. And I think it’s just [*that*] they do have a different way of doing things. They’re on a mission. So, you don’t feel like you can go interrupt them ….’ (Anja, MBCHB, DH)

Mari reflected on her hesitancy to refer to doctors actually being based on her own lack of insight into what they were up against:

‘I was hesitant to refer to an ortho because I didn’t know if it was worse enough for them to actually see. Because in public health, it’s basically this thing of, if it’s not too bad, then it’s not too bad. So you sort of need extreme cases to … justify why you want them to see the patient because I know they’re overloaded with their work.’ (Mari, PT, DH)

Differences in culture, religion and language contributed to the resistance of graduates to collaborate. This was summarised well by Mieke:

‘I think often when there’s like different, either like you say, culture, religion, all of that, like the way people do things or communicate is different. I think maybe some cultures they’re more direct, where I might be offended, but if I realise it’s a different culture, it’s not that they meant to be that direct, but that’s just the way they are.’ (Mieke, PT, DH)

The need to recognise, understand and respect differences extended beyond the medical team and included the need to understand the patient and family who also form part of the interprofessional team:

‘I think something that I realised this year is actually like how white medicine really is and it’s scary because we have like so many parents that come to us … as a last resort, which is quite scary. And then to keep the person’s culture in mind, and to have a lot of cultural humility, and not just westernise them.’ (Emile, SPT, PH)

### Theme 3: Recommendations for undergraduate health professions education

Participants reflected on their experiences and offered recommendations for UGT to promote effective IPE that may better prepare other graduates for interprofessional collaboration in the workplace.

#### Sub-theme 3.1: More contextually relevant exposure

There was a strong indication from participants that they wanted practical exposure to IPE that was integrated into UGT and contextually relevant to the current healthcare system:

‘At the university they tell you, you need to think and plan about it, but … it’s really something we need to do, being at the placement and then facilitating the process of how do you actually do this now in the reality where things aren’t that easy.’ (Isaac, PT, DC)

The importance of taking IPE outside of the classroom into practice where it can be facilitated and integrated into usual patient care was noted, rather than having a separate IPCP focus on one rotation during training:

‘In [*X rural platform*] we obviously had collaboration, but it was basically only one block that we focused on it. So I think just having it more like … not a separate thing. Like always collaborate … not only in [*X*] … everyone can have that opportunity to collaborate, I think it teaches you a lot.’ (Dala, OT, DH)

‘[*E*]ven just in like clinical, if there could be some other collaborative work at that level because you can know in theory, okay, this is what you refer to OTs for … but until you’re with the patient and they’re thinking out the box and you’re like, Oh my word, that is magnificent.’ (Rebecca, PT, DH)

Despite the many challenges that IPE faces in terms of logistics, graduates felt that with some coordination and alignment, IPE in other environments would be possible:

‘I think if you’re intentional about it. Yes [*collaboration*] works, in [*X rural platform*] they set out time for their high-risk clinics, their SAM clinics, their wheelchair clinics where it is collaborative … What’s to say you can’t do that for students in [*XX academic hospital*], it’s going to take being intentional about it. Making it a priority because your schedules are not always going to line up.’ (Rebecca, PT, DH)

In order for IPE to be coordinated and contextually relevant, graduates mentioned the need for educators to be up to date with what was happening on the ground to understand the need for IPCP:

‘I think they need … a wakeup call with regards to how the system is, I mean for example I had patients coming to me asking for SASSA [*state financial support*] referral letters. And I’m like, “what? Why? Like, what?”’ (Mari, PT, DH)

To assist lecturers in staying up to date with contextual clinical realities, Rebecca suggested:

‘[*A*] reverse mentorship model [*where lecturers learn from new graduates*], cause that’s going to impact your case studies in third year … The lecturer that’s presenting the case now has a conversation with the commserve [*graduate*] about on the ground stuff.’ (Rebecca, PT, DH)

#### Sub-theme 3.2: Communication, advocacy and role modelling in practice

Kari voiced the importance of having access to other professions in the clinical environment and interacting with them prior to graduation to learn communication skills and build relationships which also needed to be role modelled by educators:

‘You need to be able to say “I don’t 100 percent agree because of this, this and this.” That is also a skill you need to learn, if you at least have seen the doctor once or twice and greeted them, it’s much easier to approach them … you need to learn to approach with respect.’ (Kari, OT, PH)

Mieke reflected on her limited exposure to practical advocacy and communication in a real-life setting which in turn affected her confidence to collaborate:

‘What I found is you don’t know how to communicate in a way that you explain what your scope is, so I think that’s quite daunting. So, giving people kind of tips how to actually facilitate a conversation might help a lot.’ (Mieke, PT, DH)

Learning strategies to get buy-in and take initiative with practical examples, were suggested by Nicolette:

‘You need to know how to approach people and how to develop professional relationships with them. Practical things like what do you need to do to take initiative? You need to maybe make a phone call to someone who you don’t know at all. So even things like that is scary.’ (Nicolette, OT, DH)

The need for communication skills to be taught in a practical way, where students are facilitated through real encounters was reinforced by Emile’s statement where he reflected that skills development requires real-life examples, but he doubted that this was something the university could offer:

‘I think in many ways, undergrad is doing what they can with what they have. Because I think some things only experience can teach you? Like I can’t learn how to work with difficult people unless I’m actually working with difficult people.’ (Emile, SPT, TH)

However, the lack of opportunities for practical skills development relevant to IPE were reflected on by participants, especially when it related to the importance of role modelling:

‘I think it’s definitely a skill that can be taught at an undergrad level. I think everyone in the medicine and health science faculty always just says “You need to advocate.” … And when the lecturers are telling us you need to advocate, in my mind, I’m sitting in the lecture and I’m like, “Okay, my name is [*Taugheeda*]. I’m a speech therapist. This is what I do.” But actually, having … that discussion about what it is that you do.’ (Taugheeda, SPT, DH)

‘We were taken into hospitals like even before we did like the clinical blocks … they motivate us to talk to the doctors, but there was never like a facilitation or role modelling on how to do that.’ (Mari, PT, DH)

Emile noted that even the language used by educators during UGT relating to professional advocacy was defensive … rather than encouraging them to see the value of their profession and promote it:

‘Unfortunately, it’s been conditioned into us to say, “You have to get on your soapbox because we’re seen as not important” [*as speech therapists*]. That negative aspect of the sentence. But what if we just teach our students to say, in the same context, but different light, “Just remember speech is important. Speak up for yourself.”’ (Emile, SPT, TH)

#### Sub-theme 3.3: Accepting differences

One of the recommendations made to mitigate the challenges to IPCP related to profession, culture, language and religion was through exposure and acceptance that these differences exist:

‘[*Culture*] definitely has an influence and if you realise it does have an influence, then it would help … So if it’s not something you’re used to I think the uncertainty of how to now engage often makes people hesitant to communicate.’ (Mieke, PT, DH)

‘You can’t change those things [*demographics – age, culture, religious beliefs*], but those things do impact, how you engage … Because you can’t change them, the knowledge of those things can at least change your response … at undergrad teach that there’s differences and we’ve got to teach the individual how to still engage and build relationships despite those differences.’ (Rebecca, PT, DH)

Insight into other professions’ level of responsibility and the pressures they face, specifically doctors, was proposed as a means of reducing graduates’ hesitancy to engage and be valuable in establishing IPCP:

‘Every allied knows the role of a doctor, but if you maybe get an idea of the day to day decisions a doctor needs to make … to just be mindful of that … if only we understood the process and what the doctors are up against and what the problems are, then it would be easier. In undergrad, you don’t understand the stress of a doctor.’ (Kari, OT, PH)

Having direct exposure to other professions who could facilitate their learning was considered a valuable part of understanding professional responsibilities and how they could work together:

‘So if there was just … a doctor or registrar … that talked to you as a physio and answered questions … about how a usual working day goes here and their necessity for physio input. I think that would have broken the barrier for me between being a student physio now practicing as a new graduate. And just the, the reality of what they have to deal with so that we both understand from both sides where it’s coming from.’ (Mari, PT, DH)

#### Sub-theme 3.4: The value of relationships

Graduates frequently referred to the value of having established relationships with other professions during their training on the rural platform, but having longer periods to build relationships was highlighted as a way to improve IPCP after graduation:

‘So when I was final year, thanks to our rural placement, I met some amazing OTs, physios, dietetics students … especially the ones we saw socially, because we actually became friends. Then sometimes throughout the year, we’d be like, “I have this case, should I refer to OT, should I not? Like, what are your recommendations?”’ (Emile, SPT, TH)

‘Having a bit of a longer block where you get opportunity to build relationships and stay there a bit longer, I think it will really benefit people … because you get to know the doctors as well in the ward.’ (Mieke, PT, DH)

In a situation where two participants who had both spent their final year training on one of the training platforms, were placed together in a large hospital, an interesting dynamic started based on their previous acquaintanceship:

‘It’s such a hopeless department if I can say it like that … I just want some positivity, so, I was always “[*Sindiso*], let’s do something.” I felt quite bad because I referred all the mental health people to him because I know he does something about it, which is also, not fair on him.’ (Anja, MBCHB, DH)

Sindiso, however, felt grateful for the opportunity to be acknowledged and be part of a team:

‘I was actually still very close to [*Anja*], she’s been … the one that’s referring more than all of the interns. and then she would just, if I would meet her during the wards, she would ask me “what can you do with this patient?”’ (Sindiso, OT, DH)

## Discussion

Based on the findings, it is evident that aspects of existing IPE initiatives during training on the two rural platforms such as a holistic approach to patient care, previously established relationships and knowledge of other professions assisted in participants’ eagerness and ability to engage in IPCP in the workplace. However, graduates’ willingness to incorporate IPCP in the working environment and the sustainability in doing so were very different, which is not unusual in environments where IPCP is not commonplace.^4,11^

It is one thing if graduates who have not had exposure to IPE as students do not engage in IPCP, but is another thing if those who have been exposed and recognise its importance are struggling to do it. The graduates from this study were not prepared for some of the communication and hierarchy barriers they faced in initiating IPCP. This demonstrates that they were not given sufficient tools to engage in IPCP in the context in which they were expected to work.^34^ If we as educators do not align IPE with the realities of the healthcare system and the challenges that exist there, we may threaten the chances of improving and sustaining IPCP in the workplace.^13^

Health professions curricula must align IPE with local healthcare realities to prepare graduates for real-world challenges.^9,13^ Training should address barriers and facilitators in the local system, tailoring undergraduate education to graduates’ needs. Educators should understand these challenges, potentially through mentorships with new graduates.

This study highlights a gap between training and reality, showing limited exposure to interprofessional conflict and power dynamics despite opportunities for relationship development and understanding professional roles. Interprofessional education was confined to one context and the rural training period. The findings suggest a need for more practical IPE with workplace IPCP opportunities, guided by contextually aware educators.

**FIGURE 1:**
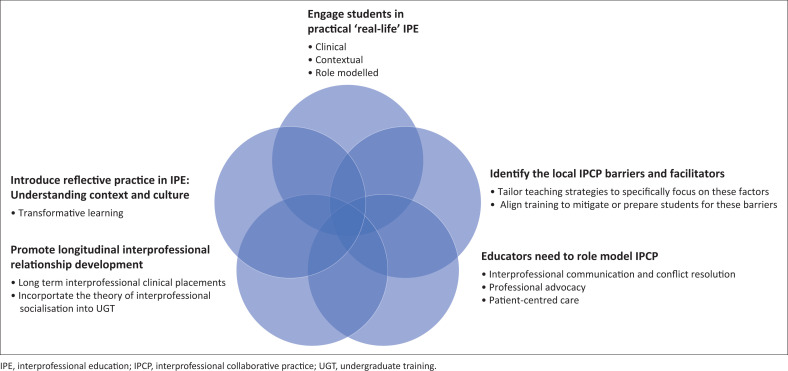
Training fit-for-purpose graduates: Practical recommendations for interprofessional education during undergraduate training to promote interprofessional collaborative practice after graduation.

We discuss next some of the practical recommendations for undergraduate IPE to promote IPCP after graduation which include the use of existing theories available in HPE. None of the theories are discussed in depth but are suggested as useful strategies to consider when trying to improve responsive IPE during UGT. The recommendations discussed are summarised in Figure 1, which shows the overlapping nature of initiatives that can be introduced during UGT to train fit-for-purpose graduates who can engage in IPCP.

### Engage in practical real-life interprofessional education

Shrader et al.^9^ demonstrated that graduates who had been exposed to IPE as students had a positive influence on IPCP in the workplace after graduation. However, the practices introduced by the graduates in this study were limited by hierarchy and professional ignorance of colleagues. Graduates were left despondent and ‘scared’ to engage in some situations because what they were experiencing was foreign to them. This was because they had had limited to no real-life experience of interprofessional conflict or how hierarchy in the working environment can affect patient care. Practical IPE that takes place in the clinical environment is most effective in connecting education to practice,^16,35,36^ but this needs to be done in real-life interprofessional patient-centred contexts that include communication challenges related to hierarchy, culture and ignorance.^13^ Cox et al.^14^ note that IPE that takes place outside of the clinical environment is considered ineffective in connecting IPE to healthcare systems outcomes. The call for contextual practice-based IPE for students is not new^37,38^ and research demonstrates that dedicated clinical IPE placements encourage openness to collaborate with other professionals after graduation.^35^ Training students in the current healthcare system, which presents enough challenging scenarios to IPCP, provides a valuable space for facilitators to model and discuss the practical implementation of IPE to students during UGT. These facilitators need to provide adequate role modelling and opportunities for reflection and discussion on what is happening during IPE. Sharing the responsibility of facilitation between educators from various programmes can alleviate the challenge of limited supervisory resources in the clinical context.

### Role modelling of interprofessional collaborative practice

Despite recognising the importance of teaching interprofessional communication skills during UGT, it remains a barrier to IPCP in the workplace.^16,39^ Graduates emphasised the need to develop these skills in practical settings with educators who role-model them. Educators and clinicians must demonstrate IPCP in both workplace and classroom, allowing students to practice these skills in context.^16,40^ Participants highlighted the need for conflict resolution and communication skills training during UGT, supported by studies showing these skills predict the ability to resolve interprofessional conflict.^41^ Health professions education should prioritise communication competence and conflict resolution in IPE to promote effective IPCP.^13^ However, competence alone is insufficient; educational approaches must shift from theory-based to practical and contextual training.^42,43^

Graduates felt they were taught to advocate for their profession from a position of undervaluation, which could perpetuate hierarchy and self-doubt. Positive and assertive role modelling should be encouraged during training. Faculty development is essential and should include interprofessional activities, conflict scenarios and modelling interprofessional patient management.^38^ Institutions must commit to upskilling educators to train and lead by example, rather than relying on self-nominated IPE champions.^16^

### Interprofessional relationship development

This study supports Roberts et al.’s^44^ claim that the longer students are placed together, the stronger the interprofessional relationships developed. Our study also shows that this makes them more likely to engage collaboratively after graduation, even remotely. Time must be spent investing in interprofessional relationship development during UGT, instead of teaching students about one another in isolation and then hoping graduates will be patient-centred and collaborative. Interprofessional socialisation (IPS) during UGT has been shown to improve IPCP^36^ and has been identified as an important process in the development of an interprofessional identity (sense of belonging to an interprofessional community).^45^ Khalili et al.^46^ provide a framework for IPS that can be used during UGT to promote the development of an interprofessional identity to promote IPCP. The use of this framework has been previously published in the context of this study.^25^

Participants noted that engaging and communicating with other professions during UGT and gaining an understanding of their work realities could have value in improving their self-confidence to collaborate. Interprofessional relationship development is crucial in the preparation of graduates for IPCP.^34^ These relationships can be facilitated through shared living spaces and transport, intentional overlapping clinical training sites^25^ and training by professionals other than their own.^47^

### Reflective practice: Understanding context and culture

Participants recommended undergraduate exposure to the perceived barriers of IPCP, even if they knew they could not be changed, for example, cultural differences. Participants had the insight to know that learning about different cultures is not enough but learning how to reflect on and accept these differences is beneficial. The understanding of cultural humility can be fostered during UGT by immersing students in their patients’ contexts for extended periods and facilitating their transformation through reflective practice.^48,49,50^ Students may also benefit from training alongside students from different professions, cultures and backgrounds who live and work together on rural platforms, gaining a deeper understanding of culture.^51^

The need to develop evaluative judgement through reflective practice is what Baradell^42^ considers essential to critique traditional knowledge systems and seek new knowledge and understanding. Reflection as a process in developing evaluative judgement is crucial as institutions of higher education cannot teach individuals every necessary knowledge to cope with the complexity of healthcare and the healthcare system. They can, however, prepare students to identify their own learning needs and develop the drive and capabilities needed to seek this growth and understanding throughout their professional lives. Transformative learning can offer a useful theory to facilitate students’ process of critical reflection after facing, for example, an uncomfortable situation such as interprofessional conflict because of cultural differences. The aim of transformative learning is for students to continuously reflect on their beliefs, assumptions and actions to expand their worldview and change their behaviour.^52^ Transformative learning should be viewed as a continuous process that institutions can facilitate in their students to equip them for the multitude of barriers to IPCP in clinical practice and for them to become the change agents we hope they will grow into.

### Limitations of the study

Despite the relatively small sample size, we believe that the transferability of our findings to some settings and contexts could be possible, because of the rich descriptions provided. Exploring the perspectives of both graduates exposed to IPE during UGT and those that were not, would have been valuable to understand other potential influencing factors that affect graduate IPCP.^7,53^

Understanding the social and contextual factors that influence IPCP in the workplace would be a valuable next step in exploring how HPE can promote IPCP in their clinical training spaces for a start. Understanding the socioecological factors that influence IPCP in SA or any country for that matter could make it easier to determine what can be done to improve it.

## Conclusion

Health professional graduates face difficulties engaging in and promoting IPCP in the workplace. This is especially true when they are faced with issues of hierarchy, professional ignorance and communication challenges. This article explores considerations for responsive undergraduate IPE curricula that provide contextually relevant interprofessional skills development such as practical advocacy and communication in the workplace. Unless students witness issues of hierarchy (seniority and professional hierarchy) during patient management, critically reflect and learn from positive role models, they may not be prepared for the realities of practice. It is possible that the effort put into undergraduate IPE and interprofessional skills development could be wasted if training is not in line with the realities of the healthcare system.

Exposure to relevant practical IPE through longer placements or continuous engagement with peers should be facilitated and encouraged as part of the formal curriculum. Responsive IPE can positively influence IPCP in the workplace and should therefore be aligned to the needs and challenges of the healthcare system.
